# System-wide targeted analysis of oxylipins and lipoproteins in chronic peripheral neuropathic pain—an explorative study

**DOI:** 10.1097/PR9.0000000000001305

**Published:** 2025-07-02

**Authors:** Mika Jönsson, Emmanuel Bäckryd, Stefan Ljunggren, Nina Ottosson, Amaia Jauregi-Miguel, Sara I. Liin, Antonio Checa, Craig E. Wheelock, Bijar Ghafouri

**Affiliations:** a Pain and Rehabilitation Centre, and Department of Health, Medicine and Caring Sciences, Linköping University, Linköping, Sweden; b Occupational and Environmental Medicine Centre in Linköping, and Department of Health, Medicine and Caring Sciences, Linköping University, Linköping, Sweden; c Division of Cell and Neurobiology, Department of Biomedical and Clinical Sciences, Linköping University, Linköping, Sweden; d Unit of Integrative Metabolomics, Institute of Environmental Medicine, Karolinska Institutet, Stockholm, Sweden; e Department of Respiratory Medicine and Allergy, Karolinska University Hospital, Stockholm, Sweden

**Keywords:** Cytokine, Inflammation, Lipoproteins, Oxylipins, Octadecanoids, Kv7.2/7.3 channel

## Abstract

**Introduction:**

Neuroinflammation and oxidative dysfunction, and their reciprocal interplay, are critically involved in the pathophysiology of chronic neuropathic pain (NeuP). Numerous studies have investigated the crosstalk between inflammatory biomolecules such as cytokines, chemokines, and neuronal cells. However, the impact of immunomodulatory lipoproteins and oxylipins in NeuP pathophysiology is far less explored.

**Methods:**

Using a combination of techniques, we uncovered altered lipoprotein composition in high-density lipoproteins (HDLs) and low-density lipoproteins (LDLs) with complementary alterations in the plasma profile of oxylipins and cytokines among patients.

**Results:**

Lower level of apolipoproteins in patient HDL and 2 isoforms of acute phase serum amyloid A (SAA) with higher levels in patients was found. The constitutively expressed SAA4 was detected in 6 isoforms in patients, but only 2 isoforms were detected in healthy controls. In LDL, lysozyme C and 2 isoforms of SAA were exclusive to patients. Analysis of protein carbonylation showed oxidation of 6 proteins in HDL, of which 3 were unique to patients. No oxidized proteins were observed in LDL. Oxylipin analysis revealed 13 octadecanoids that were significantly downregulated in patients, of which 7 demonstrated significant activating effects on the Kv7.2/7.3 channel, which is anticipated to dampen neuronal signalling in sensory afferents. Among the significant octadecanoids, 9-HODE showed most prominent facilitating effects on Kv7.2/7.3 channel activation.

**Conclusion:**

These results present a previously unexplored network of integrated alterations of lipoproteins, octadecanoids, and cytokines in patients suffering from NeuP, indicative of deviant immuno-protective functioning across several biological systems including lipid metabolic processes, inflammation, and Kv7.2/7.3 signalling.

## Abstract 2

Supplemental Digital Content is Available in the Text.

Downregulated immunomodulatory apolipoproteins/octadecanoids and upregulated proinflammatory cytokines, acute-phase serum amyloids A, and lysozyme C were found in patients. The Kv7.2/7.3 channel was significantly activated by certain octadecanoids.

## 1. Introduction

Neuropathic pain (NeuP), caused by a lesion or disease in the somatosensory nervous system, is one of the most intractable chronic pain types affecting approximately 7% to 10% of the general population.^21,24^ Although, the molecular mechanisms that drive and maintain NeuP remain to be elucidated, much evidence suggests an intrinsic relationship between immune cells and central and peripheral neuroinflammation.^24,33^

Lipoproteins present a vast array of circulating molecules of pleiotropic immunomodulatory abilities with high susceptibility to oxidative changes. Various oxidative modifications can be introduced to lipoproteins by numerous reactive agents; eg, reducing sugars, reactive oxygen species (ROS), lipid peroxidation–derived aldehydes, and protein carbonylation, which is a hallmark of oxidative damage.^2^ During inflammation, high-density lipoproteins (HDLs) regularly lose some of its protective functions among which its ability to protect low-density lipoproteins (LDLs) from oxidation.^3,41^ Oxidation of lipoproteins triggers proatherogenic and proinflammatory pathways where even minimally modified LDLs can induce massive proinflammatory effects, in eg, macrophages, including cytoskeletal rearrangements, generation of ROS, and expression of inflammatory genes.^59^ The main target for oxidizing lipoproteins include apolipoproteins and polyunsaturated fatty acids (PUFAs).^4^

Oxylipins are a diverse class of specialized signalling molecules that are derived from both enzymatic and nonenzymatic oxidation of PUFAs as well as other fatty acids, see Supplements, Supplementary Figure 1, http://links.lww.com/PR9/A326.^23^ Some oxylipins have been shown to exert pro- and antinociceptive properties,^16^ particularly those derived from linolic acid, ie, octadecanoids.^52^ Apart from nociceptive properties, these oxygenated metabolites can also act as potent mediators of inflammation and exert proresolving activities.^22,28,54^

Oxylipins are formed by 3 main pathways: cyclooxygenases, lipoxygenases, and cytochrome P450s and can be classified according to the length of the carbon chain of the precursor fatty acid (eg, octadecanoids [C-18], eicosanoids [C-20], and docosanoids [C-22]).^50^ Several preclinical studies have shown that oxylipins may act as endogenous proalgesic ligands affecting the onset and maintenance of pain by directly sensitizing transient receptor potential (TRP) channels or indirectly by activating G-protein–coupled receptors.^7,44–46,56^ The family of TRP channels has been extensively studied when it comes to oxylipins and nociception where the pathological activity is associated with thermal hyperalgesia and mechanical allodynia.^14,25,48,49,56,67^ Hence, to seek other contributing mechanisms, we turned to the voltage-gated potassium channel Kv7.2/7.3, which plays an important role in regulating sensory and nociceptive signalling by reducing neuronal excitability.^17,39^ The Kv7.2/7.3 channel is expressed in sensory afferents and contributes greatly to setting the negative resting membrane potential of neurons, and to the repolarization and afterhyperpolarization phases of the neuronal action potential.^47^ Accordingly, endogenous activation or inhibition of the Kv7.2/7.3 channel by oxylipins could have regulatory functions over pain signalling by affecting the threshold for action potentials firing. The Kv7.2/7.3 channel has been previously shown to be modulated by other endogenous lipids^27,30,35–37^; however, the effect of oxylipins remains unstudied.

Subsequently, the aim of this study was to explore a system-wide targeted profile of immunomodulatory molecules at different layers of integrated systems between patients with chronic peripheral NeuP and healthy controls (HC), concurrently. As a secondary aim, the effect of oxylipins was assessed on the Kv7.2/7.3 channel using automated patch clamp (APC) electrophysiology.

## 2. Methods

### 2.1. Patients

The patient population has been described previously and will only be briefly presented here.^5^ In short, patients with chronic peripheral NeuP enrolled in a clinical trial of intrathecal bolus injection of ziconotide (ClinicalTrials.gov identifier NCT01373983) were asked for participation. All samples were collected before any administration of the analgesic drug. For inclusion, the following criteria had to be met: ≥18 years of age; chronic (ie, ≥3 months) peripheral NeuP subsequent to surgery or trauma where conventional pharmacological treatment had been ineffective; Visual Analogue Scale Pain Intensity ≥ 40 mm^19^; cognitively capable; and written informed consent. After signing informed consent, a medical examination was performed, and the following data were registered: pain diagnosis; pain duration; present and past medical history; and concomitant medication. All patients had, according to criteria published by Treede et al.,^58^ at least probable post-traumatic/postsurgical neuropathic pain. See Table 1 for basic demographic data over patients with NeuP and HC included in respective analysis and Table 2 for NeuP patient characteristics.

**Table 1:** Basic demographic data over patients with neuropathic pain and healthy controls included in respective analysis.

2DE and HDL oxidation	Patients (n = 9)	Healthy controls (n = 2)
Sex (female %)	44.4	0
Age (mean ± SD)	59.7 ± 8.6	24.5 ± 4.9
BMI (mean ± SD)	26.6 ± 4.3	22.9 ± 0.7
VASPI (mean ± SD)	76.3 ± 9.8	NA

Oxylipin analysis	Patients (n = 16)	Healthy controls (n = 12)
Sex (% female)	43.7	58.3
Age (mean ± SD)	59.8 ± 9.1	42.8 ± 15.3
BMI (mean ± SD)	26.6 ± 3.6	23.9* ± 2.9
VASPI (mean ± SD)	71.6 ± 13.1	NA

Oxylipin and cytokine analysis	Patients (n = 7)	Healthy controls (n = 6)
Sex (% female)	42.8	50.0
Age (mean ± SD)	54.6 ± 5.1	42.8 ± 14.9
BMI (mean ± SD)	27.3 ± 4.3	24.3 ± 2.5
VASPI (mean ± SD)	74.0 ± 17.3	NA

* Mean BMI for healthy controls was based on 10 instead of 12 subjects due to missing information from 2 healthy controls.

2-DE, 2-dimensional gel electrophoresis; BMI, body mass index; HDL, high-density lipoprotein; NA, not applicable; SD, standard deviation; VASPI, visual analogue scale for pain intensity last week.

**Table 2:** Neuropathic pain patient characteristics.

ICD 10	VASPI (0–100 mm)	Pain duration (mo)	Comorbidities
S342 and G629	75	120	History of alcohol dependency; psoriasis; tension type headache
S342	72	39	Polymyalgia rheumatica; hypertension
S740	82	120	Orthostatism; vertebral compressions; vitreous detachment
S549	64	300	None
S949	60	58	None
S342	72	72	Psoriasis; hypertension
S342	59	60	Mild angina; mild obstructive lung disease
S342	87	36	Hypertension; anaemia; dyspepsia
S342	40	120	None
S343	78	79	None
G629	68	78	None
S142	58	12	Fibromyalgia
S342	83	48	Localized bladder tumour
S342	74	120	Depression
S142	84	18	Hypertension

S142, injury of nerve root of cervical spine; S342, injury of nerve root of lumbar and sacral spine; S549, injury of unspecified nerve at forearm level; S740, injury of sciatic nerve at hip and thigh level; S949, injury of unspecified nerve at ankle and foot level; G629, polyneuropathy, unspecified; VASPI, visual analogue scale for pain intensity last week. ICD-10, International Classification of Diseases, 10^th^ Revision.

### 2.2. Healthy controls

Recruitment was conducted by local advertisement at the Faculty of Medicine and Health Sciences, Linköping University Sweden. Written informed consent was necessary for participation after which participating subjects underwent a structured interview designed to ensure absence of any significant medical condition preventing inclusion. See Table 1 for demographic data.

#### 2.2.1. Ethics

The study was approved by the Ethical Review Board Regional Ethics Committee in Linköping, Sweden (Dnr M136-06 and Dnr 2012/94-32) and followed the Helsinki Declaration and Good Clinical Practice. All participants were given verbal and written information, and written informed consent was collected from participating subjects before data collection.

#### 2.2.2. Sample collection

A 10-mL sample of peripheral venous blood was collected in an ethylenediaminetetraacetic acid (EDTA) tube. Drawn samples were immediately transferred to Painomics laboratory, Linköping University Hospital, where each sample was centrifuged, aliquoted in 500 μL volume, and stored in −86°C. One aliquot was thawed and used for each analysis. Laboratory analyses were conducted on site in the Painomics Laboratory, the research laboratory of the Liin Group, and by the Chemical Biology Consortium Sweden, Linköping University Hospital. Oxylipin analyses were conducted in the Small Molecule Mass Spectrometry Core Facility, Karolinska Institute, Stockholm.

### 2.3. Biochemical analysis

#### 2.3.1. Cytokines and chemokines in plasma

The analysis of cytokines and chemokines have been described previously and will only be briefly mentioned here.^27^ The concentration of 71 cytokines and chemokines was investigated in plasma from patients with chronic peripheral NeuP and HC using a customizable U-PLEX assay based on an electrochemiluminescent detection method (Meso Scale Diagnostics, Rockville, MD). Data acquisition and analysis was conducted using MESO QUICKPLEX SQ 120 instrument equipped with DISCOVERY WORKBENCH data analysis software (Meso Scale Diagnostics). Cytokine raw data are listed in Supplements, Supplementary Table 1, http://links.lww.com/PR9/A326.

#### 2.3.2. Lipoprotein isolation

Density-gradient ultracentrifugation was used to isolate LDL and HDL from EDTA plasma as described previously with minor modifications.^31,32^ The isolation protocol is detailed in Supplements, http://links.lww.com/PR9/A326.

#### 2.3.3. Proteome profile analysis of low-density lipoprotein and high-density lipoprotein

In short, 200 μg of LDL or 240 μg of HDL were separated in the first dimension by isoelectric focusing on 11-cm immobilized pH gradient (IPG) strips (pH 3-10) at 32000 vHr (max 8000 V) overnight. After derivatization and equilibration of the IPG strips, the proteins were separated in the second dimension horizontally on the HPE BlueHorizon (SERVA) using 2D HPE Large Gel (nonfluorescent film backing [NF] 12% kit, SERVA), the 2-dimensional gel electrophoresis (2-DE) protocol is detailed in Supplements, http://links.lww.com/PR9/A326.

#### 2.3.4. Analysis of oxidized proteins

Identification of oxidized LDL and HDL was conducted by electroblotting proteins separated by 2-DE or 1-DE to polyvinylidene fluoride membranes using the Trans-Blot Turbo System (Bio-Rad Laboratories, Hercules, CA). The derivatized carbonyl group with 2,4-dinitrophenylhydrazine was detected using anti-DNP antibody (Sigma-Aldrich, Darmstadt, Germany), for details see Supplements, http://links.lww.com/PR9/A326.

#### 2.3.5. Analysis of oxylipins

Oxylipins were extracted and determined as previously published^34^ with some modifications. The extraction and UPLC-MS protocol, as well as specific chromatographic and MS parameters used for each oxylipin are detailed in Supplements, http://links.lww.com/PR9/A326.

#### 2.3.6. Kv7.2/7.3 QPatch recordings

The stably expressing cell line Chinese hamster ovary (CHO)-hKv7.2/7.3 (B'SYS GmBH, Witterswil, Switzerland) was used to study the cells' currents on the APC system QPatch II 48 (Sophion Bioscience A/S, Ballerup, Denmark). The APC protocol, as well as information about cell culture, compounds and analysis of ion current data are detailed in Supplements, http://links.lww.com/PR9/A326.

### 2.4. Statistical analysis and bioinformatics

Statistical analyses were conducted in IBM SPSS (version 24.0; IBM Corporation, Route 100, Somers, NY) and SIMCA-P+ (version 17.0; Sartorius Stedim Biotech, Umeå, Sweden). For descriptive analysis, IBM SPSS was used. The nonparametric Mann–Whitney *U* test was used for comparisons between groups. Results were visualized using SIMCA-P+, GraphPad Prism 10, PDQuest.

For data with low subject-to-variable ratios, where variables had high degrees of intercorrelations, advanced multivariate data analysis by projection was applied using SIMCA-P+ following recommendations presented by Wheelock and Wheelock.^63^ First, relevant data were isolated from background noise by unsupervised principal component analysis (PCA), followed by Hotelling T2 and distance to model in X-space (DModX) for detection of potential multivariate outliers. To regress class belonging, orthogonal partial least squares discriminant analysis (OPLS-DA) was used. As previously described, the OPLS-DA analysis was finalized in 2 steps where oxylipins and/or cytokines with an absolute *P* (corr) > 0.4 and a variable influence on projection (VIP) ≥ 1 were used in a second regression model. The correlation coefficient *P* (corr) is comparable between models and is generally considered significant if absolute *P* (corr) ≥ 0.3. The VIP value signifies the importance of each variable to the model, and a VIP value ≥1.0 was deemed significant. The results from the second model, ie, the new R^2^ (goodness of fit), Q^2^ (goodness of prediction), and analysis of variance of cross-validated predictive residuals (CV-ANOVA), were presented.

For ion channel data, mean values were expressed as mean ± SEM. For comparisons between compound and run-specific control, a 2-way ANOVA test (Gaussian distribution of residuals and equal SDs assumed) with Dunnett multiple comparisons test was used.

#### 2.4.1. Network analysis

The association network of protein–protein interactions between identified HDL/LDL-related proteins from 2-DE analysis and cytokines was conducted using the online database Search Tool for Retrieval of Interacting Genes/Proteins (STRING; version 12).^57^ Details over the network analysis are presented in Supplements, http://links.lww.com/PR9/A326.

## 3. Results

### 3.1. Proteome profile of high-density lipoprotein and low-density lipoprotein

**Figure 1.**
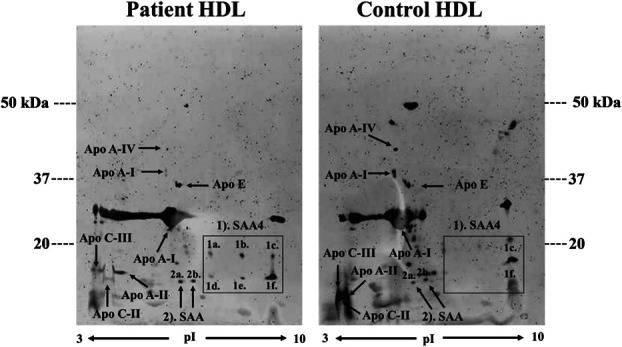
Protein pattern in a master gel of pooled samples of isolated HDL from patients with NeuP and healthy controls. HDL (240 μg) isolated from plasma was separated with 2-DE and stained with Lumitein. Visually, patient HDL differed in several aspects compared to healthy control HDL, ie, less Apo A-I, Apo A-II, Apo A IV, Apo C-II, Apo C-III. In patient HDL, 6 isoforms of serum amyloid IV were detected, shown by the black box (1a–f), compared to healthy control where only 2 isoforms were spotted (1c and 1f). Two isoforms of SAA1 (2a–2b) were seen in both patient and healthy control HDL. 2-DE, 2-dimensional gel electrophoresis; HDL, high-density lipoprotein; SAA, serum amyloid A.

**Figure 2.**
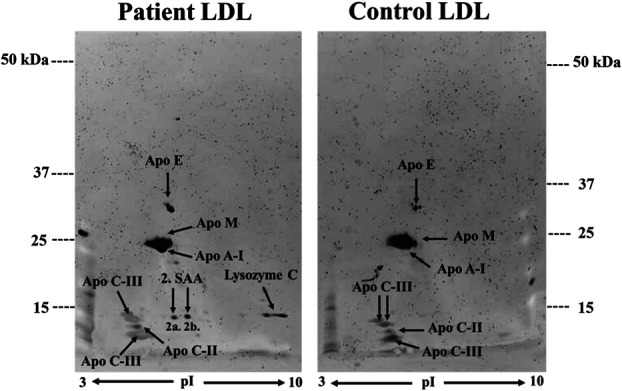
Protein patterns in a master gel of pooled samples of isolated LDL in patients with NeuP and healthy controls. LDL isolated from plasma was separated with 2-DE and stained with Lumitein. Patient LDL showed 2 isoforms of serum amyloid A1 (2a–b) and lysozyme C, which was absent in healthy controls. Visually, apolipoprotein: Apo M, Apo A-I, Apo C-II, and apo C-III all appeared to be less intense in LDL from patients compared to healthy controls. 2-DE, 2-dimensional gel electrophoresis; LDL, low-density lipoprotein.

The HDL protein pattern revealed several differences between patients and HC according to the pooled samples (Fig. 1). For instance, patient HDL appeared to contain less of Apo A-I, Apo A-II, Apo A IV, Apo C-II, Apo C-III, but more serum amyloid A (SAA) when visually compared to HC (Fig. 1). Furthermore, 6 isoforms of SAA4 were detected in patient HDL (1a–f), whereas only 2 isoforms (1c and 1f) were found in control HDL. Spot number 1a–c of SAA4 was most likely glycosylated isoforms of spots 1d–f as described earlier by Karlsson et al.^31^ In LDL, serum amyloid A and lysozyme C were only detected in patients (Fig. 2). Moreover, apolipoproteins: Apo M, Apo A-I, Apo C-II, and apo C-III all appeared to be less intense in LDL from patients compared to HC (Fig. 2).

### 3.2. Oxidation of proteins in plasma

#### 3.2.1. Two-dimensional gel electrophoresis oxidation in high-density lipoprotein but not in low-density lipoprotein

**Figure 3.**
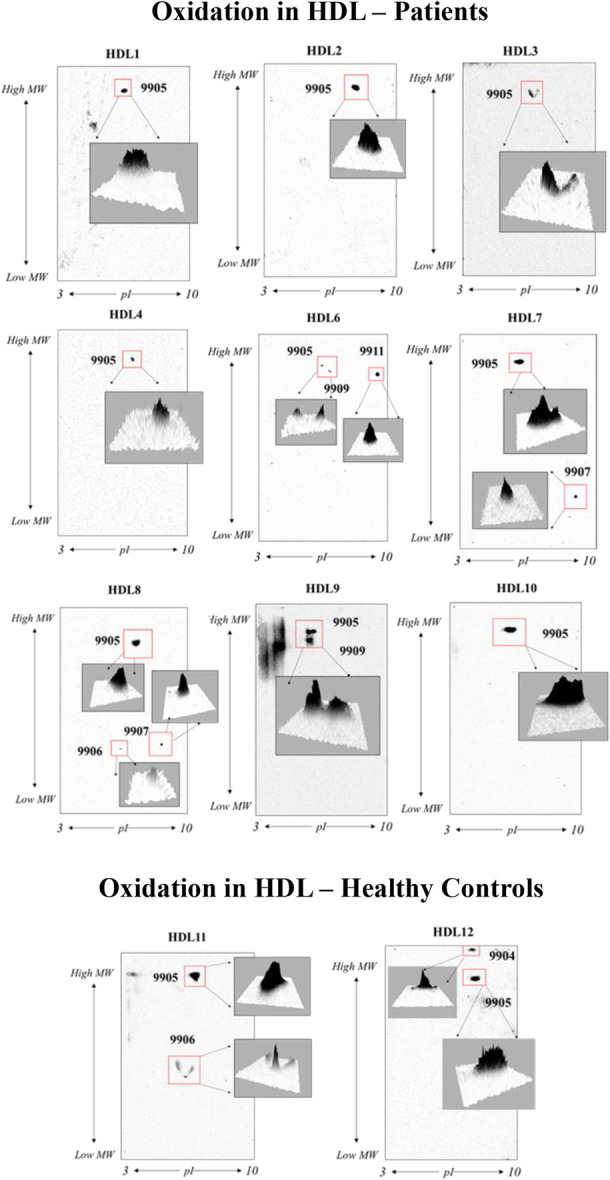
Oxidation of HDL proteins after 2-DE. Six oxidation spots were identified (9904, 9905, 9906, 9907, 9909, 9911) where spots 9907, 9909, and 9911 were unique for patients with NeuP. Spot 9905 was detected in all samples, whereas 9906 was found in one patient sample (HDL8) and one healthy control (HDL11). Spot 9904 was only detected in one healthy control (HDL12). The marked areas are shown in three-dimensional view. Patient samples were HDL1-HDL10 and healthy controls HDL11-12. High MW—50 kDa. 2-DE, 2-dimensional gel electrophoresis; HDL, high-density lipoprotein. MW, molecular weight.

There were no oxidized proteins that could be detected in LDL; however, 6 oxidized protein spots (9904, 9905, 9906, 9907, 9909, 9911) were found in samples of HDL (Fig. 3). Spot N^o^ 9905 was detected in all samples and most likely represented albumin. The spot N^o^: 9907, 9909, and 9911 were unique for patients with NeuP, whereas Spot N^o^ 9904 was only present in 1 healthy control (HDL12). Spot N^o^ 9907 corresponded to the localization of SAA4 and were present in 2 patients (HDL7 and HDL8), whereas spot N^o^ 9909 and 9911 were found in one patient (HDL6) and were most likely isoforms of albumin. Similarly, spot N^o^ 9904 was also likely an isoform of albumin. Spot N^o^ 9906 was found in one patient sample (HDL8) and one healthy control (HDL11) and was localized in the area corresponding to Apo A-I (Fig. 3).

### 3.3. Identification of class discriminating oxylipins in plasma

**Figure 4.**
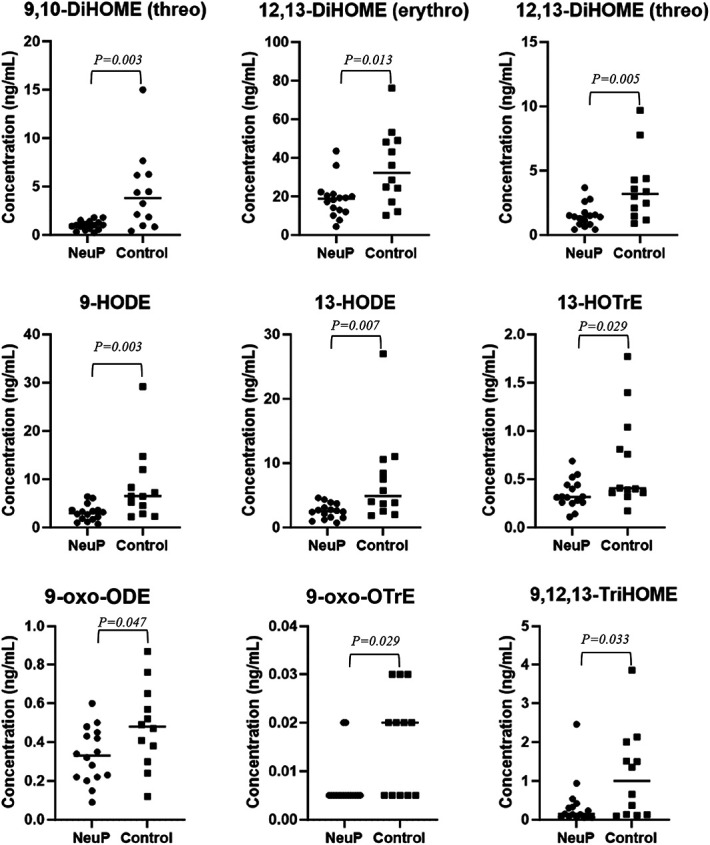
Concentration of significant octadecanoids for group discrimination by Mann–Whitney *U*. Each dot represents a patient or healthy control, the median value is depicted by a horizontal line.

A significant OPLS-DA regression model for differences in oxylipin concentration between patients with NeuP (n = 16) and HC (n = 12) was obtained, 41 X-variables, R^2^ = 0.419, Q^2^ = 0.339, and *P* = 0.006 by CV-ANOVA. Table 3 lists 13 octadecanoids that were significant for the OPLS-DA model, and Figure 4 depicts the concentration of 11 oxylipins that were significant for group separation indicated by Mann–Whitney U.

**Table 3:** List of 13 octadecanoids that were significant for the orthogonal partial least squares discriminant analysis model.

Oxylipin	*P* (corr)	VIP
9,10-DiHOME (*THREO*)	0.87	3.05
13-HODE	0.87	3.03
9-HODE	0.86	2.99
13-HOTrE	0.84	2.92
9-HOTrE	0.81	2.84
9,10,13-TriHOME	0.80	2.81
12,13-DiHOME (*THREO*)	0.80	2.78
9-oxo-OTrE	0.76	2.65
12(13)-EpOME (*CIS*)	0.71	2.49
9(10)-EpOME (*CIS*)	0.64	2.22
9-oxo-ODE	0.59	2.05
9,12,13-TriHOME	0.58	2.04
12,13-DiHOME (*ERYTHRO*)	0.46	1.60

All octadecanoids were downregulated in patients with NeuP compared to healthy controls, as indicated by a positive *P* (corr). The VIP value signifies the importance of each variable to the model.

NeuP, neuropathic pain; VIP, variable influence on projection.

### 3.4. Regression of class-discriminating oxylipins and cytokines in plasma

**Figure 5.**
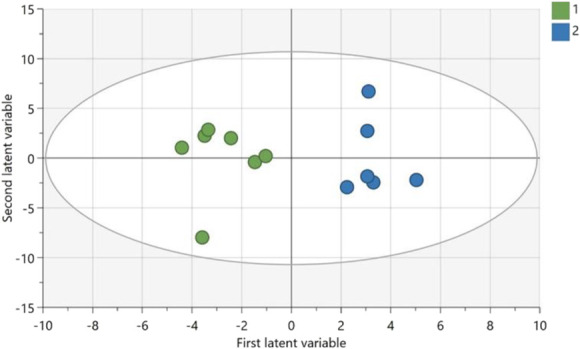
Score plot of OPLS-DA regression model of oxylipins and cytokines. The x-axis corresponds to interclass separation, and the y-axis corresponds to the first orthogonal component. Each dot represents a patient (green) or healthy control (blue). OPLS-DA, orthogonal partial least squares discriminant analysis.

**Figure 6.**
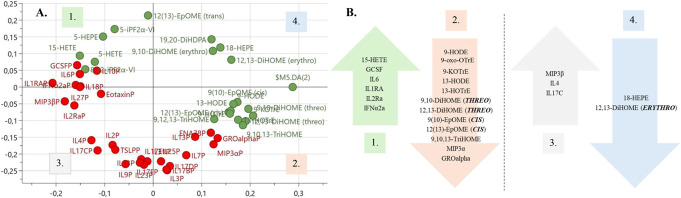
(A) Loading plot of cytokines and oxylipins associated with the OPLS-DA regression model. The loading plot mirrors the score plot in Figure 5, showing the intercorrelation between cytokines and oxylipins within the model. Variables depicted close to each other are positively correlated, whereas variables located on opposite sites diagonally demonstrate an inverse relationship. Cytokines/oxylipins on the left are more strongly associated with patients with NeuP, whereas substances on the right are more related to healthy controls. Oxylipins are depicted in green and cytokines in red. (B) Schematic drawing of (A) illustrating the inverse relationship between significant oxylipins and cytokines depicted in Table 3. Each arrow represents one quadrant from (A) (indicated by the corresponding number 1–4) and is listed with its associated cytokines and oxylipins. The left-hand side of the vertical line illustrates the inverse relationship between cytokines and oxylipins located between the upper left (quadrant no 1) and lower right (quadrant no 2) diagonal in (A), whereas the right-hand side illustrates the inverse relationship between cytokines and oxylipins located between the lower left (quadrant no 3) and upper right (quadrant no 4) diagonal in (A). IL6 was not significant to the model but was added out of interest due to previous findings, both by our lab and others, suggesting its importance to NeuP.^29^ OPLS-DA, orthogonal partial least squares discriminant analysis.

To explore the multivariate interplay between cytokines and oxylipins in NeuP, an OPLS-DA regression model of class-discriminating cytokines and oxylipins between patients (n = 7) and HC (n = 6) was computed, 112 X-variables, R^2^ = 0.902, Q^2^ = 0.738, and *P* = 0.02 by CV-ANOVA (Fig. 5). The analysis was restricted to patients and HC whose inflammatory profile had previously been investigated and thus had cytokine data as well as oxylipin data.^29^ A distinct separation between patients and HC was noted (Fig. 5). Table 4 lists the 22 cytokines and oxylipins that were significant for the OPLS-DA model. To explore the intercorrelation between cytokines and oxylipins within the model, a loading plot was created, illustrating the inverse relationship between the significant oxylipins and cytokines (Fig. 6).

**Table 4:** List of 22 oxylipins and cytokines significant for the orthogonal partial least squares discriminant analysis regression model shown in Figure 5.

Oxylipin/Cytokine	VIP	*P* (corr)
9,10-DiHOME (*THREO*)	1.38	0.76
IL1RA	1.36	−0.76
9-oxo-OtrE	1.31	0.72
12,13-DiHOME (*THREO*)	1.30	0.70
9,10,13-TriHOME	1.27	0.68
MIP3β	1.20	−0.67
13-HOTrE	1.20	0.64
9(10)-EpOME (*CIS*)	1.15	0.64
12,13-DiHOME (*ERYTHRO*)	1.10	0.59
9-HODE	1.10	0.61
13-HODE	1.08	0.59
IL2Ra	1.08	−0.60
9-HOTrE	1.08	0.58
12(13)-EpOME (*CIS*)	1.06	0.57
GCSF	1.06	−0.58
IFNα2a	1.05	−0.59
15-HETE	1.05	−0.55
GROalpha	1.03	0.49
MIP3α	1.02	0.46
IL17C	1.02	−0.42
IL4	1.02	−0.47
18-HEPE	1.01	0.51

The VIP value signifies the importance of each variable to the model. A negative *P* (corr) indicates higher levels in patients with NeuP compared to healthy controls and vice versa.

NeuP, neuropathic pain; VIP, variable influence on projection.

### 3.5. Specific octadecanoids facilitate activation of the neuronal Kv7.2/7.3 channel

**Figure 7.**
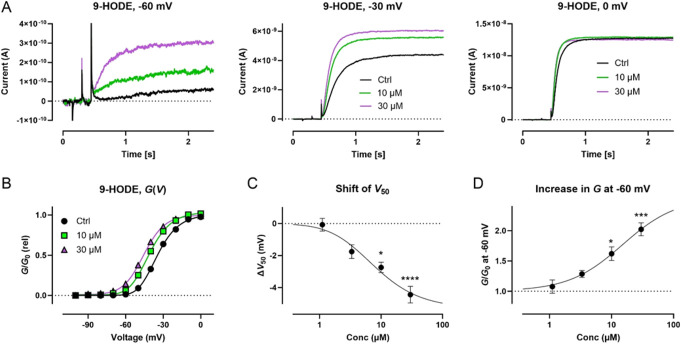
9-HODE effects on the Kv7.2/7.3 channel. (A) Representative example of the ability of 9-HODE to increase current amplitude at indicated voltages. Black traces represent control (only vehicle added), green represent 10 µM of 9-HODE, and purple represent 30 µM of 9-HODE. (B) Corresponding G (V) curve for the representative example shown in (A). The curves are fits to Equation 1. Best fit for ctrl: V50 = −34.6 mV. Best fit for 10 µM: V50 = −40.9 mV. Best fit for 30 µM: V50 = −45.6 mV. The slope was 8 for all fits. (C–D) Concentration–response relationship for the 9-HODE effect on V50 (C) and G at −60 mV (D). Data shown as mean ± SEM. n = 7. Statistics denote 1-way ANOVA followed by Dunnett multiple comparison test. The curves are fit to equation 2. Best fit for V50: top = −5.3 mV, Kd = 3.3 µM, N = 2. Best fit for G at −60 mV: top = 2.5, Kd = 15.1 µM, N = 1.

To assess putative octadecanoid effects on the Kv7.2/7.3 channel, we used automated patch-clamp electrophysiology and CHO cells stably expressing the human Kv7.2/7.3 channel. Octadecanoid effects on the voltage dependence of channel opening (*V*_50_) and the maximal conductance (*G*) were quantified as described in the Materials and Methods. We found that 7 of the 13 significant octadecanoids shown in Table 3 demonstrated significant activating effects on the Kv7.2/7.3 channel, seen as a hyperpolarizing shift in *V*_50_ and/or an increase in *G* (Table 5). For 9-HOTrE, 9,10-DiHOME (*threo*), 13-HODE, 12,13-DiHOME (*threo*), 9-oxo-ODE, and 9(10)-EpOME (*cis*), the activating effects were seen at the highest tested concentration (30 µM). However, for 9-HODE, clear activating effects were seen already at 10 µM, with a significant shift in *V*_50_ of −2.7 ± 0.3 mV, which increased to −4.4 ± 0.5 mV at 30 µM (Table 5). This hyperpolarizing shift in *V*_50_ allows the channel to open at more negative voltages and, hence, generate larger currents at a large range of voltages. Figure 7 shows a representative example of the ability of 9-HODE to increase the Kv7.2/7.3 channel current amplitude at different voltages (Fig. 7A) and to shift *V*_50_ towards more negative voltages (Fig. 7B). Figure 7C summarizes the concentration–response relationship for the *V*_50_ effect of 9-HODE. Similarly, Figure 7D summarizes the concentration–response relationship for the ability of 9-HODE to increase channel conductance near the resting membrane potential of neurons (−60 mV). Altogether, these experiments show that specific octadecanoids facilitate Kv7.2/7.3 channel activation by shifting *V*_50_ and/or increasing *G*, with 9-HODE showing most prominent effects.

**Table 5:** Electrophysiological recordings of significantly reduced octadecanoids in patients with neuropathic pain (Table 3).

Oxylipin species	1.11 µM		3.33 µM		10 µM		30 µM		n
	Δ*V*_50_ (mV)	*G*/*G*_0_ (rel)	Δ*V*_50_ (mV)	*G*/*G*_0_ (rel)	Δ*V*_50_ (mV)	*G*/*G*_0_ (rel)	Δ*V*_50_ (mV)	*G*/*G*_0_ (rel)	
9,10-DiHOME (*THREO*)	0.49 ± 0.53	1.03 ± 0.03	0.19 ± 0.6	1.09 ± 0.05	0.01 ± 0.9	1.17 ± 0.08	−0.25 ± 1.12	1.21 ± 0.13*	8
13-HODE	−0.39 ± 0.24	1.02 ± 0.01	−1.29 ± 0.67	1.01 ± 0.02	−1.65 ± 1.02	1.03 ± 0.03	−2.18 ± 0.92*	1.01 ± 0.06	8
9-HODE	−0.07 ± 0.4	1.02 ± 0.01	−1.75 ± 0.43	1 ± 0.03	−2.74 ± 0.33*	1.01 ± 0.03	−4.44 ± 0.52***	1 ± 0.04	7
13-HOTrE	0.02 ± 0.76	0.98 ± 0.06	0.89 ± 0.75	1.09 ± 0.05	−0.24 ± 0.98	1.1 ± 0.07	−0.17 ± 0.88	1.12 ± 0.07	6
9-HOTrE	−0.44 ± 0.41	0.99 ± 0.03	−1.18 ± 0.38	1 ± 0.04	−1.84 ± 0.45	0.98 ± 0.05	−2.93 ± 0.65**	1.05 ± 0.06	8
9,10,13-TriHOME	1.47 ± 0.78	0.91 ± 0.03	−0.7 ± 1.34	0.91 ± 0.02	−1.17 ± 1.42	1.07 ± 0.02	−1.2 ± 1.74	1.19 ± 0.04	5–6
12,13-DiHOME (*THREO*)	−0.1 ± 0.19	1 ± 0.03	−0.99 ± 0.28	1.06 ± 0.04	−0.04 ± 0.62	1.12 ± 0.04	0.04 ± 0.65	1.21 ± 0.04*	8
9-oxo-OTrE	0.25 ± 0.47	1 ± 0.06	−1.15 ± 1.01	1.03 ± 0.07	−0.9 ± 1.09	1.06 ± 0.08	−1.01 ± 1.08	1.1 ± 0.09	7
12(13) -EpOME (*CIS*)	0.02 ± 0.39	1 ± 0.02	0.03 ± 0.4	1.02 ± 0.04	−0.42 ± 0.4	1.02 ± 0.08	−1.98 ± 0.92	1.11 ± 0.13	7–8
9(10) -EpOME (*CIS*)	−0.17 ± 0.56	1 ± 0.02	−0.65 ± 0.84	1.04 ± 0.03	−2.9 ± 0.75	1 ± 0.02	−3.83 ± 0.6**	1 ± 0.1	6–7
9-oxo-ODE	−0.57 ± 0.35	1.02 ± 0.03	−1.27 ± 0.56	1.04 ± 0.05	−1.9 ± 0.82	1.02 ± 0.07	−3.28 ± 0.66**	0.98 ± 0.07	7–8
9,12,13-TriHOME	2.66 ± 0.36	0.82 ± 0.04	−0.21 ± 0.65	0.93 ± 0.09	0.31 ± 1.34	1.11 ± 0.14	0.24 ± 1.19	1.23 ± 0.2	6
12,13-DiHOME (*ERYTHRO*)	−0.1 ± 0.69	0.98 ± 0.03	0.11 ± 0.73	1.1 ± 0.06	0.97 ± 0.86	1.06 ± 0.06	0.78 ± 1.13	1.05 ± 0.11	5–6

Δ*V*_50_ and *G*/*G*_0_ determined as described in the methods section. Data shown as mean ± SEM. Statistics calculated using 2-way ANOVA and Dunnett multiple comparisons test. n indicates the number of experiments (see Methods). Significant values are highlighted in bold.

*P < 0.05, **P < 0.01, ***P < 0.001.

### 3.6. Network analysis

**Figure 8.**
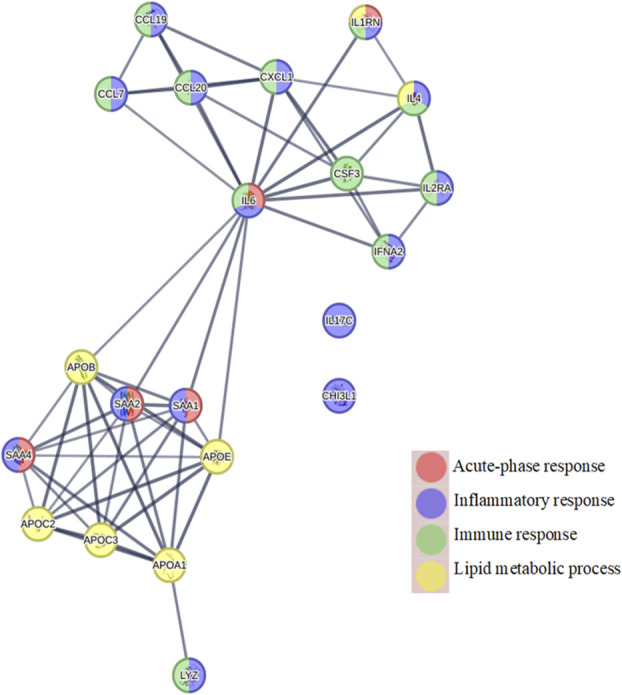
Network analysis: illustrating the relationship between identified HDL/LDL-associated proteins from 2-DE analysis together with cytokines important for regression of class-discriminating oxylipins and cytokines Table 4. Three previously identified cytokines important for class discrimination (42) were added (IL6, CCL7 (MCP3), CHI3L1 (YKL-40)). CCL19 (MIP3β), CCL20 (MIP3α), CXCL1 (GROalpha), CSF3 (GCSF), IL1RN (IL1RA), and IFNA2 (IFNα2A). 2-DE, 2-dimensional gel electrophoresis; HDL, high-density lipoprotein; LDL, low-density lipoprotein.

The protein–protein association network and biological processes involved, according to Gene Ontology, was investigated between identified HDL/LDL-related proteins from 2-DE and cytokines significant for the OPLS-DA regression model (Fig. 5). The resultant protein–protein interaction (PPI) network revealed significantly more interactions than expected, meaning that the integrated proteins had more interactions amongst themselves than what would be expected for a random set of proteins of similar size drawn from the genome (PPI enrichment *P*-value: <1.0e–16, Fig. 8). Thus, indicating that the included proteins were at least partially biologically connected as a group. Biological processes included from Gene Ontology were acute-phase response, inflammatory response, immune response, and lipid metabolic process. Two clusters of proteins were clearly formed in the network, connected centrally by IL6. Only IL17-C and CHI3L1 did not show any interactions in the network.

## 4. Discussion

Several differences were seen in the proteome profile of HDL and LDL between patients with NeuP and HC. In HDL, the main apolipoprotein, Apo A-I, which constitutes 70% of total HDL protein content,^60^ appeared downregulated in patients compared to HC (Fig. 1). Apolipoprotein A-I is mostly known for its cardioprotective functions, but recent studies suggest additional therapeutic roles in cancer, diabetes, rheumatoid arthritis, sepsis, and neurological disorders.^10,42^ Many of the reported beneficial and cardioprotective effects of Apo A-I revolves around inflammation. For instance, lipid-free Apo A-I has been shown to act as an anti-inflammatory molecule by inhibiting the activation of proinflammatory macrophages leading to downstream suppression of proinflammatory cytokine production.^10^ Similarly, Apo A-II, the second most abundant apolipoprotein in plasma HDL, constituting 20% of total HDL, together with Apo A-IV, Apo C-II, and Apo C-III, all appeared to be downregulated in patients (Fig. 1).

In contrast, the acute-phase protein SAA1 was more abundant in plasma HDL from patients with NeuP compared to HC (Fig. 1). Serum amyloid A is one of the principal acute-phase proteins in humans and elevates dramatically in concentration after inflammation. Upon secretion to the blood circulation, SAA binds to HDL and displaces Apo A-I leading to HDL saturation and high levels of free circulating SAA.^11,13^ The displacement of Apo A-I by SAA reduces the anti-inflammatory functions of HDL and hampers its ability to mediate reverse cholesterol transport from macrophages.^42^ Moreover, SAA is chemotactic for monocytes, neutrophils, mast cells, and T cells and can act to induce autocrine and paracrine cytokine and chemokine production in various cell types along with activating the inflammasome cascade.^13^ Notably, although SAA bound to HDL impairs the anti-inflammatory properties of HDL, the association also dampens the proinflammatory functions of SAA, which are acclaimed to lipid-free SAA.^55^ For instance, HDL has been shown to inhibit SAA-mediated ROS generation and NLRP3 inflammasome activation.^55^ Apart from hepatic synthesis, SAA can also be extra-hepatically produced at local sites of tissue inflammation by eg, macrophages and fibroblast-like synoviocytes, where its concentration appears to correlate with the severity of the inflammatory disease.^9^ Accordingly, elevated SAA has been implicated in several inflammatory diseases including atherosclerosis, rheumatoid arthritis, type 2 diabetes, and Crohn disease.^65^ As such, SAA exhibits significant immunomodulatory activities and has been shown to activate multiple receptors to exert its immunological functions, ie, Toll-like receptor 2 and 4 (TLR2 and TLR4), formyl peptide receptor-like 1 (FPRL1), FPRL2, the ATP receptor P2X7, CD36, and the scavenger receptor SR-BI.^20,55,65^ The present result of lower levels of Apo A-I in patients with NeuP is consistent with both our current result of higher SAA as well as previous results that showed an inflammatory profile of upregulated cytokines and chemokines in plasma and saliva from the same cohort of patients with NeuP.^29^ Similarly, network analysis of protein–protein interactions showed strong relationships between serum amyloids and apolipoproteins, where SAA1 and SAA2 further interact with IL6 (Fig. 8). Thus, collectively these results point in the direction of aberrant functioning of HDL in NeuP, both in altered apolipoprotein composition and increased SAA associated HDL, both of which affecting cytokine homeostasis and inflammation.

Likewise, apolipoproteins in patient LDL appeared less expressed compared to HC. However, only 2 isoforms of SAA were spotted together with the bacteriolytic enzyme lysozyme C, exclusively in patients (Fig. 2). Interestingly, the gene expression of lysozyme has been shown to be upregulated in neurodegenerative disorders and nerve injuries during sterile neuroinflammation.^62,64^ Since there was no associated bacterial load, the biological bacteriolytic function of upregulated lysozyme was presumably without cause, thus questioning its physiological role in NeuP.^64^ However, it has been shown in rodent models that endogenously upregulated lysozyme can act on neuronal TLR4 promoting neuronal hyperexcitability and consequently neuropathic pain-like behaviour without engaging other immune cells.^64^ Network analysis linked lysozyme C to Apo A-I, indicating biologically meaningful interactions (Fig. 8).

Carbonylation analysis after 2-DE showed no oxidized proteins in LDL while several oxidized proteins in HDL were found (Fig. 3). Since Apo B-100 is a high-molecular-mass protein (>400 kDa), it is typically not shown in 2-DE separations, explaining why Apo B-100 oxidation was not captured in LDL after 2-DE (see Supplements, Supplementary Figure 2, http://links.lww.com/PR9/A326). In HDL, 6 oxidized protein spots were identified where 3 protein spots were unique for patients corresponding to SAA4 and albumin (Fig. 3).

Analysis of oxylipins revealed significantly downregulated levels of primarily octadecanoid lipid mediators among patients with NeuP compared to HC (Table 3). Octadecanoids are 18-carbon containing oxylipins that are derived from dietary PUFAs including linoleic and alpha linolenic acid.^50^ These compounds have previously been linked to nociception^16^ with the hypothesis that high dietary linoleic acid is associated with chronic pain.^51^ In addition, linoleic acid–derived octadecanoids have been reported to regulate neuronal morphogenesis in rats^12^ and neurovascular-glial disruption.^66^

Interestingly, 7 of 13 octadecanoids that were significantly reduced in patients with NeuP also demonstrated significant activating effects on the Kv7.2/7.3 channel. Since the Kv7.2/7.3 channel is predominately expressed in nociceptive dorsal root and trigeminal ganglion neurons, with loss of function leading to neuronal membrane hyperexcitability, it possess potential regulatory roles in nociception.^1^ Accordingly, it has been suggested that functional downregulation of Kv7 channels contribute to peripheral sensitization of nociceptors, and thus a potential underlying mechanisms of NeuP pathophysiology.^38^ Moreover, retigabine, a potent Kv7.2/7.3 channel activator, has been shown to alleviate NeuP behaviour in several animal models, with effectiveness similar to gabapentin, a first line drug for NeuP.^6,15^ Hence, lower concentration of specific oxylipins among patients with NeuP could potentially cause less Kv7.2/7.3 channel activation, which might contribute to enhanced pain signalling due to diminished endogenous protective function.

The linolic acid derivatives, 9-HODE and 13-HODE, both demonstrated Kv7.2/7.3 facilitating effects, where 9-HODE was most prominent (Table 5, Fig. 7). These linolic acid derivatives, specifically 13-HODE, have been reported as regulators of inflammation in various cell systems and diseases for instance in metabolic syndrome and cancer.^40,61^ The beneficial effects appear to be mediated by HODEs ability to decrease interleukins (ILs) and NOS as well as increasing HDL.^61^ For instance, it has been reported that 9-HODE and 13-HODE inhibit the release of IL-6 from human monocytes^53^ and that exercise-induced oxidative changes are consistent with higher plasmatic 9-HODE and 13-HODE and inversely lower plasmatic IL6 and granulocyte-colony stimulating factor (GCSF).^43^ This inverse relationship between HODEs and IL6 purportedly demonstrate a regulatory function of HODEs, as they can attenuate the inflammatory response at sites of injury, possibly by activating PPAR-γ.^53^ Interestingly, this inverse relationship between HODEs, IL6, and GCSF was also shown in this study (Fig. 6). This finding suggests that lower levels of HODEs could cause aberrant regulation of inflammation at sites of injury or disease, thereby contributing to increased systemic levels of IL6 and GCSF in patients with chronic peripheral NeuP.

The soluble epoxide hydrolase (sEH)-derived products of linoleic acid, 9,10-DiHOME(*threo*) and 12,13-DiHOMEs(*threo*/*erythro*), as well as the 9,10-DiHOME precursor 9(10)-EpOME(*cis*) were all significantly downregulated in patients (Fig. 4). Interestingly, sEH inhibitors (sEHI) are currently an attractive analgesic target for NeuP as well as inflammatory-related diseases, because they block the enzyme from hydrolysing the epoxyeicosatrienoic acids (EETs) to the corresponding vicinal diols, which are potent endogenous analgesic metabolites.^8,18,26^ It has been suggested that the DiHOMEs may display additional or unique activities in a pathophysiological state compared to their precursor epoxylipids.^67^ For example, it has been shown that 12,13-DiHOME induces thermal pain hyperalgesia during inflammatory pain via transient receptor potential vanilloid 1 (TRPV1) activation.^67^ Here, we showed that 9,10-DiHOME(*threo*), 12,13-DiHOME(*threo*), and 9(10)-EpOME(*cis*) also have activating effects on the Kv7.2/7.3 channel, which conversely suggests potential analgesic properties (Table 5).

### 4.1. Limitations

There are several limitations in this study. The small sample size and unfortunate demographic gap between the groups depended on the fact that this was an additional study conducted from samples collected to a prior ziconotide trial. Hence, the number of patients were calculated with respect to outcomes in that trial. The ziconotide trial included cerebrospinal fluid sampling, and the invasive nature of the procedure limited the inclusion of both gender- and age-matched HC. For the HDL and LDL proteome analysis, it is important to keep in mind that the electropherogram results are based on a pool of samples taken from a limited number of subjects and neither intra- nor interindividual variations of protein expressions are known at present. For the electrophysiological analysis, another limitation concerns the difficulties of translating circulating plasma concentrations to local oxylipin concentrations near neurons and neuronal ion channels.

## 5. Conclusion

Here, we present a network of highly interconnected protein constituents of HDL and LDL, cytokines, and oxylipins, accentuating aberrant functioning in several biological processes in NeuP. An inverse relationship between lower levels of protective immunomodulatory apolipoproteins/octadecanoids and upregulated proinflammatory cytokines, acute-phase SAAs, and lysozyme C was found in patients with NeuP, a pattern which was not seen in HC. Similarly, 7 of 13 significantly reduced octadecanoids also showed significant activating effects on the Kv7.2/7.3 channel, possibly contributing to enhanced peripheral sensitization of nociceptors due to diminished Kv7.2/7.3 activation. Combined, these results present a previously unexplored network of integrated alterations of lipoproteins and oxylipins in patients with NeuP, highlighting a potential role of lipid metabolic processes in NeuP and could possibly present novel therapeutic targets.

## Disclosures

The authors report no conflicts of interest. The funders had no role in the design of the study or in the collection, analyses, or interpretation of data, in the writing of the manuscript, or in the decision to publish the results.

## Appendix A. Supplemental digital content

Supplemental digital content associated with this article can be found online at http://links.lww.com/PR9/A326.
